# Luciferase complementation for cellular assays beyond protein–protein interactions

**DOI:** 10.1007/s44211-025-00730-y

**Published:** 2025-02-18

**Authors:** Genki Kawamura, Takeaki Ozawa

**Affiliations:** Department of Chemistry, School of Science, The University of Tokyo, 7-3-1 Hongo, Bunkyo-Ku, Tokyo, 133-0033 Japan

**Keywords:** Luciferase, Protein complementation assays, Bioluminescence, Biosensors

## Abstract

Luciferase complementation assays have emerged in 2001 as a useful tool to analyze biological processes through diverse biological assays such as cellular studies and in vivo imaging. The assay has an advantage of wide dynamic ranges, high signal-to-noise ratios, and capability for real-time monitoring of dynamic biological events with a readout of bioluminescence. While it was initially harnessed for detecting protein–protein interactions, biosensors based on luciferase-fragment complementation have achieved significant advancements in their designs, expanding versatility and applicability beyond the initial scope. This review aims to provide a comprehensive overview of designing strategies employed in split luciferase complementation assays and to highlight their diverse bioanalytical applications. Because simple bi-molecular detection of protein–protein interactions by this approach is well-established, this review will focus on introducing diverse sensor designs using the concept of split luciferase complementation.

**Graphical abstract:**

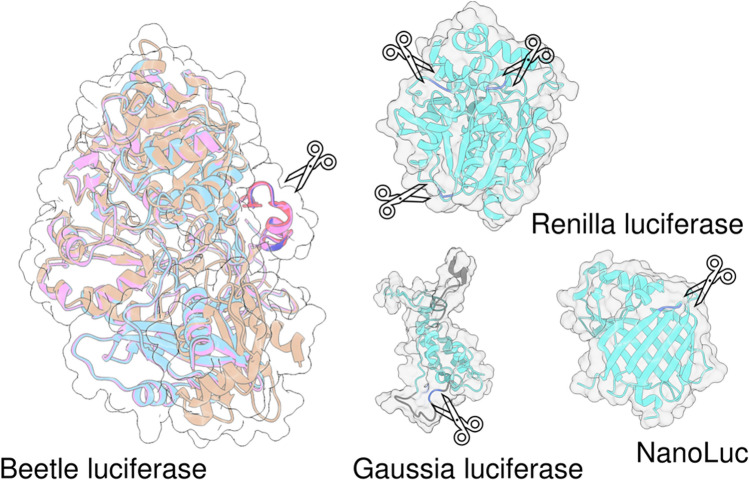

## Introduction

Protein-fragment complementation assays (PCAs) are a unique experimental technique to quantitatively assess the dynamics of protein–protein interactions (PPIs). Since the pioneering development of a conditional association of split protein fragments of ubiquitin [1], the principle of PCA has been adapted to a variety of the reporter proteins, including fluorescent protein [2], dihydrofolate reductase (DHFR) [3], *β*-lactamase [4], and bioluminescence protein such as luciferases [5, 6]. To implement a PCA, a protein is divided into two or three fragments at specific cleavage sites, rendering it an inactive state where the readout from the reporter cannot be detected. These fragments are then fused to proteins of interest. Upon interaction with the fused proteins, the fragments reassemble and regain their activity, generating a detectable signal.

The PCAs with a luciferase offer several advantages over other reporter protein-based systems. Like the conventional luciferase-based assay systems including reporter gene assays and two-hybrid systems, the luciferase complementation approach detects readouts of bioluminescence that have the character of high signal-to-noise (S/N) ratio and wide dynamic ranges [7]. This is because bioluminescence assays emit light without needing an external light source, resulting in minimal background noise, even for subtle signals. In addition, luciferase complementation assays offer an additional advantage for detecting PPIs, such as an ability to monitor them in real time. Most of the luciferase complementation assay systems are shown to be reversible, with luciferase activity switching on and off in response to changes in the complementation status of the fragmented luciferase. Furthermore, in vitro assays indicated that the time required for the bioluminescence signal to be detected upon PPI induction falls on the order of seconds [8], demonstrating that luciferase complementation assays as an ideal tool for real-time monitoring of dynamic interactions.

Over the past two decades, the system has proven its versatility in detecting various PPIs across cell-free systems, cultured cells, and in various organisms including small animals and plants. In addition, luciferase complementation biosensors have been demonstrated for their utility for high-throughput screening, real-time monitoring, and in vivo imaging of biological functions. Building upon this foundation, we will review creative design approaches and recent advancements in luciferase complementation-based biosensors. In this review, we refer to biosensors as genetically encoded probes that consist of a reporter protein, for example, a luciferase, and a sensing domain, which modulates the signal of the reporter protein in response to PPIs or binding to a target molecule.

## Basics of luciferase-fragment complementation assays

### Classification of luminescence emitting luciferase systems

Luciferases are a diverse family of enzymes that catalyze the oxidation of specific substrates, known as luciferins. This oxidative reaction excites the resulting oxyluciferin molecule, which then releases energy in the form of light as oxyluciferin returns to its ground state. The simplicity of the luciferase reaction is that it only requires the luciferase enzyme, its specific substrate luciferin, and a few cofactors for the emission of light. This feature makes the luciferase-luciferin system a versatile tool to be applied for various applications.

A wide variety of organisms on Earth, including insects, marine life, fungi, and bacteria, exhibit bioluminescence. While luciferases and luciferins have not been characterized for all bioluminescent species, currently identified luciferases can be broadly categorized based on their substrate specificity. Beetle luciferases, which utilize D-luciferin as their substrate, are found in terrestrial organisms. Marine luciferases, on the other hand, employ different substrates, ranging from reduced form of flavin mononucleotide (FMNH_2_) and a long-chain aldehyde for bacterial luciferases, tetrapyrrole for dinoflagellate luciferase, ostracod luciferin for cypridina luciferase, and coelenterazine (CTZ) for various luciferase from diverse organisms [9].

Among these, beetle and CTZ-utilizing marine luciferases are widely applied to biological assays (Table 1). Beetle luciferases generally exhibit larger protein sizes and lower luminescence signal compared to marine luciferases. However, beetle luciferases often exhibit a glow-type emission, where the catalytic activity of luciferase persists for a certain period, enabling the monitoring of sustained biological processes for over 4 days [10]. On the other hand, marine luciferases tend to have smaller sizes of 13–36 kDa for the catalytic activity, with flash-type bright emission making them a suitable luciferase for applications such as high throughput screening or subcellular localization analysis. We will explore their specific characteristics in the following sections.

**Table 1:** Properties of commonly used luciferases

Luciferase	Original species	Luciferin compound	Size (kDa)	λ_max_ (nm)	Cofactor	Refs.
*Beetle luciferases*						
FLuc	*Photinus pyralis*	D-Luciferin	61	560	ATP, Mg^2+^, O_2_	[102]
CBG	*Pyrophorus plagiophthalamus*	D-Luciferin	61	540	ATP, Mg^2+^, O_2_	[74]
CBR	*Pyrophorus plagiophthalamus*	D-Luciferin	61	615	ATP, Mg^2+^, O_2_	[74]
ELuc	*Pyrearinus termitilluminans*	D-Luciferin	61	538	ATP, Mg^2+^, O_2_	[103]
Akaluc	Engineered from FLuc	Akalumine	61	650	ATP, Mg^2+^, O_2_	[91]
Railroad-worm luciferase	*Phrixotrix hirtus*	D-Luciferin	61	628	ATP, Mg^2+^, O_2_	[12]
*Marine luciferases*						
RLuc	*Renilla reniformis*	Coelenterazine	36	480	O_2_	[104]
GLuc	*Gaussia princeps*	Coelenterazine	20	473	O_2_	[105]
OLuc	*Oplophorus gracilirostris*	Coelenterazine	106	454	O_2_	[106]
NanoLuc	Engineered from OLuc	Furimazine	19	460	O_2_	[19]
MLuc	*Metridia longa*	Coelenterazine	17	480	O_2_	[107]
TurboLuc	Engineered from MLuc	Coelenterazine	16	480	O_2_	[20]
ALuc (ALuc23)	Engineered from *Copepoda* luciferase database	Coelenterazine	21	503	O_2_	[21]
picALuc	Engineered from ALuc	Coelenterazine	13	482	O_2_	[22]

### Split-luciferase complementation of beetle luciferases

**Fig. 1:**
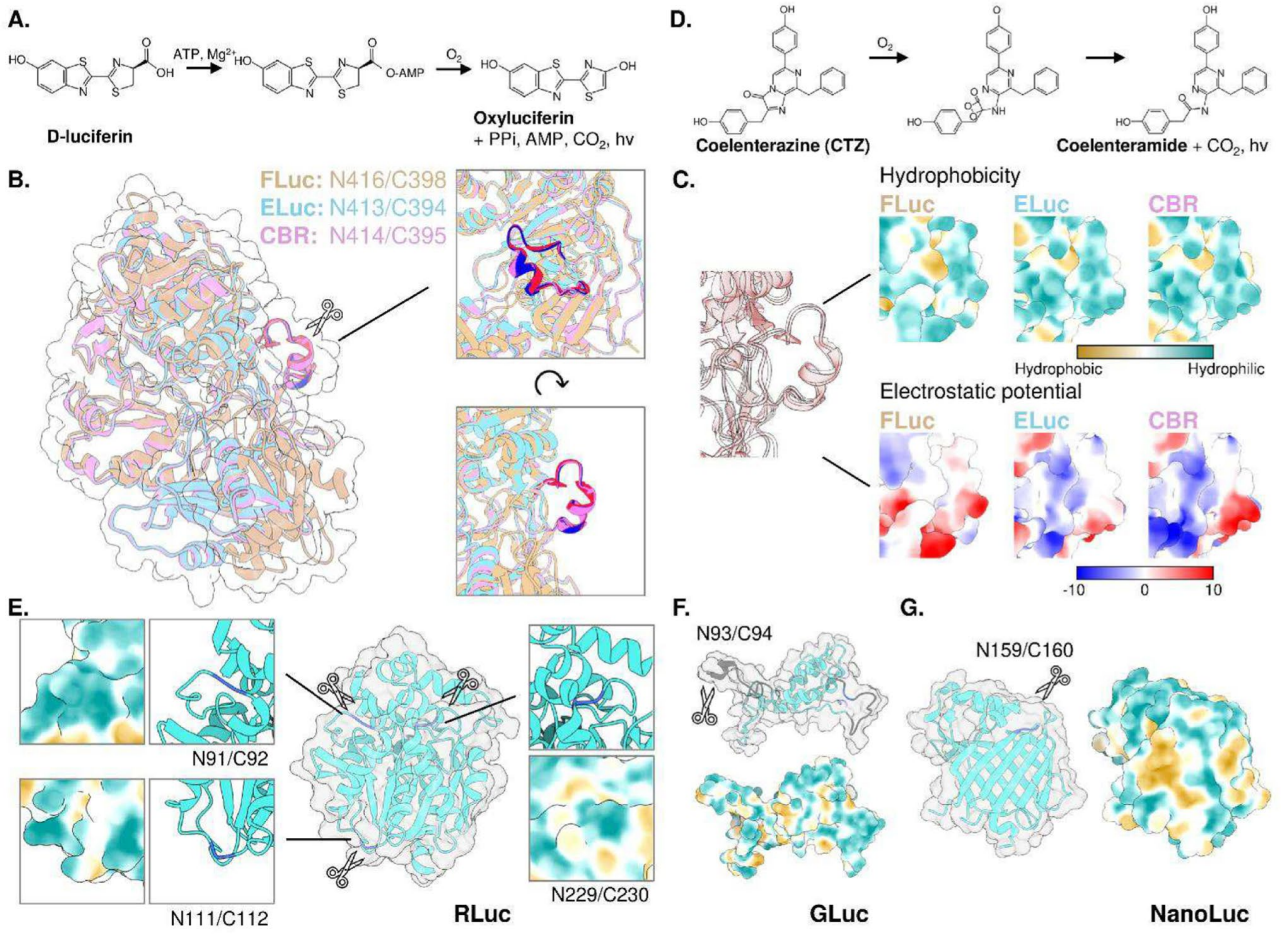
Protein structures of commonly used luciferases and their representative split points. **A** Luciferase-luciferin reaction scheme for beetle luciferases. **B** Overlapping residues for beetle luciferases. Fluc: orange, PDB: 5DV9. ELuc: sky blue, CBR: red purple. Structures of ELuc and CBR were precited using ColabFold [101]. **C** Comparison of hydrophobicity and electrostatic potential among beetle luciferases. **D** Luciferase-luciferin reaction scheme for marine luciferases. **E–G** Split point and hydrophobicity around the split point for Renilla luciferase (**E**, PDB: 2PSF), Gaussia luciferase (**F**, PDB: 9FLA), and NanoLuc (**G**, PDB: 5IBO)

Beetle luciferases, exemplified by North American firefly luciferase (FLuc, 61 kDa) from *Photinus pyralis*, catalyze the oxidation of D-luciferin in the presence of ATP, Mg^2+^, and O_2_ (Fig. 1A). Despite sharing a common substrate and homologous luciferase structure, beetle luciferases exhibit a wide range of emission spectra, spanning from green to red. For instance, blue-shifted Brazilian firefly luciferase from *Amydetes vivianii* emits at 538 nm [11], while red-shifted railroad worm luciferase from *Phrixothrix hirtus* emits at 628 nm as its emission maximum [12]. The molecular basis for the color-tuning mechanism has been extensively studied from the aspects of biochemistry and structural biology, however, because luciferase reaction involves multiple reaction steps involving various light-emitting intermediates, a comprehensive understanding of the color-tuning mechanism is yet to be identified [13].

Beetle luciferases are composed of a large N-terminal domain and a small C-terminal domain connected by a linker region of approximately 5 amino acids. Structural studies have revealed that the active site of the luciferase is primarily located within the N-terminal domain, with one crucial lysine (K529 for FLuc) in the C-terminal domain, which is involved in the phosphorylation of the intermediate during the acylation of D-luciferin [13]. This structural arrangement suggests that the composition of both domains plays a role in substrate binding and color tuning, highlighting the importance of careful split point selection.

Early attempts to develop beetle luciferase complementation assays utilized the split fragment pair of N-terminal from the start to 436 amino acid residue and C-terminal from 437 amino acid residue to the last residue (N436/C437), assuming that it would separate the N-terminal and C-terminal domains. While this approach successfully monitored interactions between IRS-1 and SH2 domain upon insulin stimulation, the N-terminal fragment retained some bioluminescent activity, even without the C-terminal domain [5]. Further research emphasized the significance of overlapping amino acids for efficient complementation in beetle luciferases (N416/C398), which was unexpectedly identified during comprehensive screening for the split-site that would reduce the background luminescence by shortening the N-terminal fragment [14]. Since then, split-point determination for luciferases has adopted a screening approach, often involving the construction of small libraries to systematically evaluate various combinations of N-terminal and C-terminal fragments [15]. Continuous efforts had successfully identified dissection sites for most commonly used beetle-type luciferases in biological studies, including click-beetle luciferases ELuc (N413/C394) [16], CBG (N413 or N414/C395) [17], CBR(N413 or N414/C395) [16, 17], and FLuc derived Akaluc (N416/C417) [18]. These insights into split points of beetle luciferase suggest a high degree of conservation, indicating that split site of the beetle luciferase could be generalized as a linker region connecting N and C terminals with an overlapping sequence (Fig. 1B). Moreover, analysis of biophysical properties across beetle luciferases revealed that regions surrounding successful split sites tend to be hydrophilic, suggesting that split site favors exposed hydrophilic regions (Fig. 1C). It is worth noting that the assay system used in the Akaluc split point determination was conducted with constitutively dimer forming proteins without considering overlapping residues. Then, split sites with overlapping regions may also exist for Akaluc that would be explored in the future study.

### Split luciferase complementation of marine luciferases

Marine luciferases utilizing CTZ as a substrate are a diverse group of enzymes that catalyze the oxidation of CTZ to produce bioluminescence (Fig. 1D). Unlike beetle luciferases, marine luciferases require only molecular oxygen (O_2_) as a cofactor. Various luciferases have been isolated from a range of marine organisms, including the sea pansy *Renilla reniformis* (RLuc, 36 kDa), the copepods *Gaussia princeps* (GLuc, 20 kDa), *Metridia longa* (MLuc, 17 kDa), and the deep-sea shrimp *Oplophorus gracilirostris* (OLuc, 106 kDa). For marine luciferases, artificially modified version of the luciferase, including NanoLuc (19 kDa) from OLuc [19], TurboLuc (16 kDa) from MLuc [20], and artificial luciferases ALuc (21 kDa) or its smaller version picALuc (13 kDa) [21, 22], has been exploited as a luciferase with favorable properties, such as improved thermostability.

Marine luciferases have also been employed as tools for analyzing biological events through split luciferase complementation strategies. Unlike beetle luciferases, marine luciferases often have a single-domain structure. Popular marine luciferases, such as RLuc, GLuc, and NanoLuc, possess well-defined split sites without overlapping amino acids (Fig. 1E-G). This suggests that complementation reassembles the fragments into their original domain structure, and excess amino acids could interfere with this association.

RLuc has been adapted for complementation assays with splits at positions N91/C92 [23], N111/C112 [24], or N229/C230 [25]. GLuc, with a split at position N93/C94 [26], has been utilized for in vivo ligand-receptor binding studies, expanding the potential applications of marine luciferases. The smallest of these systems is the split NanoLuc system, widely known as the NanoBiT system, which uses an N-terminal 159-amino acid fragment (LgBiT) and a C-terminal 11-amino acid fragment (SmBiT) to create a complementation reporter. With the coevolution of NanoLuc substrate, furimazine [19], split NanoLuc assays have been considered as one of the luciferase complementation assay systems with the most stable, brightest, and smallest in size as a tag among marine luciferases.

### Computational approach to designating the split point of luciferases

For designing a PCA, the identification of optimal dissection points is crucial. Ideally, the split sites should minimally disrupt the protein's structure, avoiding misfolding and degradation of the fragments. While spontaneous complementation can be advantageous for certain applications, such as detecting transient weak protein interactions or if the assay system is intended to be an all-or-none type detection, it can hinder the detection of dynamics of PPIs. Therefore, the split fragments would favor minimal spontaneous complementation to ensure a low background signal for detecting specific protein interactions. Conventionally, a labor-intensive screening approach has been adopted to determine split points. To circumvent this, computational approaches have been explored to predict appropriate split points. Early studies focused on unstructured regions that divide proteins into subdomain fragments, as these sites were considered less likely to disrupt the protein's structure and generate unstable fragments [14]. A similar strategy involves identifying highly hydrophilic regions that are exposed to the aqueous environment through a hydrophilic search [27].

More recently, the computational program has been designed to identify optimal split points for target proteins based on the “split energy”, a score of the total energies of the split parts relative to the native energy of the intact protein, along with solvent accessibility and sequence conservation [28]. Indeed, according to the program provided through the web interface, the program has successfully identified suitable split points for FLuc as N435/C436 (using PDB: 5DV9 as a template), which was adjacent to the originally identified split point for FLuc, N436/C437 [5]. Strikingly, three of the predicted split site of the Renilla luciferase, N88/C89, N112/C113, and N227/C228 (using PDB: 2PSF as a template), were all in close proximity to the experimentally identified split site for the RLuc (N91/C92 [23], N111/C112 [24], N229/C230 [25]), suggesting that the program can practically predict the region of sequence where optimal split site exists. However, the tool could not predict luciferases with intrinsically disordered regions such as GLuc or well-defined single domain structures such as NanoLuc, and prediction of split sites with overlapping residues is currently out of the scope of the program, possessing a necessity for further development of a program for generalized applicability.

## Design strategies for various modes of luciferase complementation

**Fig. 2:**
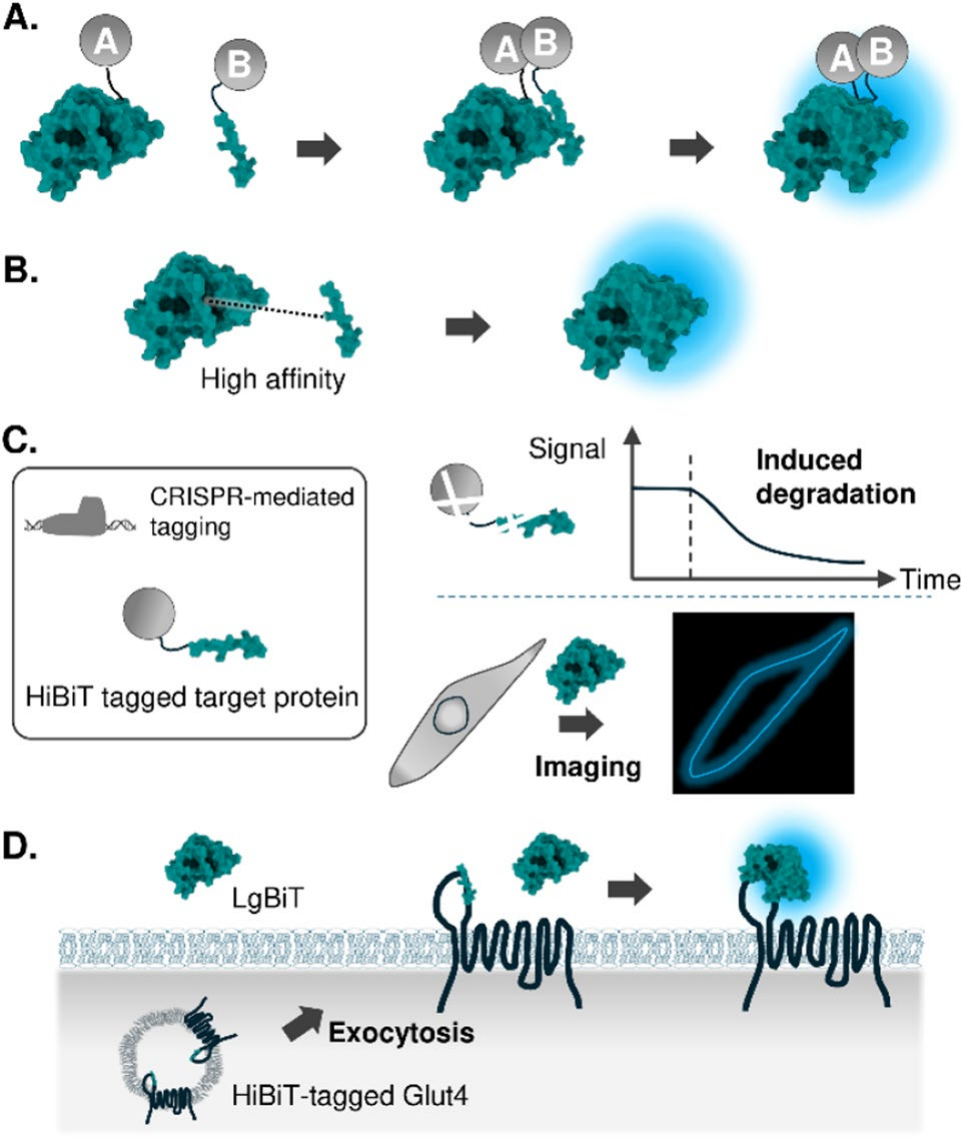
Luciferase complementation-based biosensors. **A** Schematic of the PPI-induced luciferase complementation. **B** Schematic of the spontaneous luciferase complementation using HiBiT-tag. **C** Applications of spontaneous complementation for endogenous protein dynamics analysis. **D** Quantification of transporter exocytosis using spontaneous complementation

Since the development of luciferase complementation assays, the system has been adapted to analyze a wide variety of PPIs, which has been reviewed elsewhere [29, 30]. In the following sections, we will herein introduce bioluminescence biosensors that are dependent on luciferase complementation, but that differ from simple detection of PPIs (Fig. 2A).

### Spontaneous complementation

Although luciferase complementation assays offer a promising approach for designing a system to detect biological events, enzymatic capabilities of complementation luciferase does not fully recover, making the system 0.5–10% dimmer compared to the activity of full-length luciferase [30]. To circumvent this situation, spontaneous complementation driven by a high-affinity variant of the luciferase fragment was adopted to induce stable interactions [31]. This system utilizes a small, high-affinity C-terminal fragment of NanoLuc, known as HiBiT, which binds to its partner, LgBiT, with a dissociation constant of 700 pM (Fig. 2B) [31]. This strong affinity enables spontaneous binding and signal generation. Because of its small size (11 amino acids, 1.3 kDa), HiBiT can serve as a versatile protein tag for various applications, including CRISPR-mediated tagging of endogenous proteins [31], quantitative immunoprecipitation [32], quantification of secreted chemokines [33], and quantification of degradation kinetics of a protein (Fig. 2C) [34]. With a bright emission of bioluminescence from NanoLuc, endogenous proteins that are tagged with HiBiT enabled analysis of not only their quantity but also their cellular localization [35]. Moreover, the HiBiT system can be employed to quantify cell surface protein levels. In this technique, HiBiT is fused to the outer plasma membrane exposed domain of the receptor. Membrane impermeable LgBiT is added to the system, triggering spontaneous complementation of luciferase and subsequent emission of the signal that correlates with the membrane-expressed HiBiT-tagged receptor. This strategy has been successfully applied to quantify both the endocytosis of G-protein coupled receptor (GPCR) [36], and also an exocytosis of glucose transporter type 4 (Glut4) (Fig. 2D) [37].

### Tripartite split-luciferase complementation

**Fig. 3:**
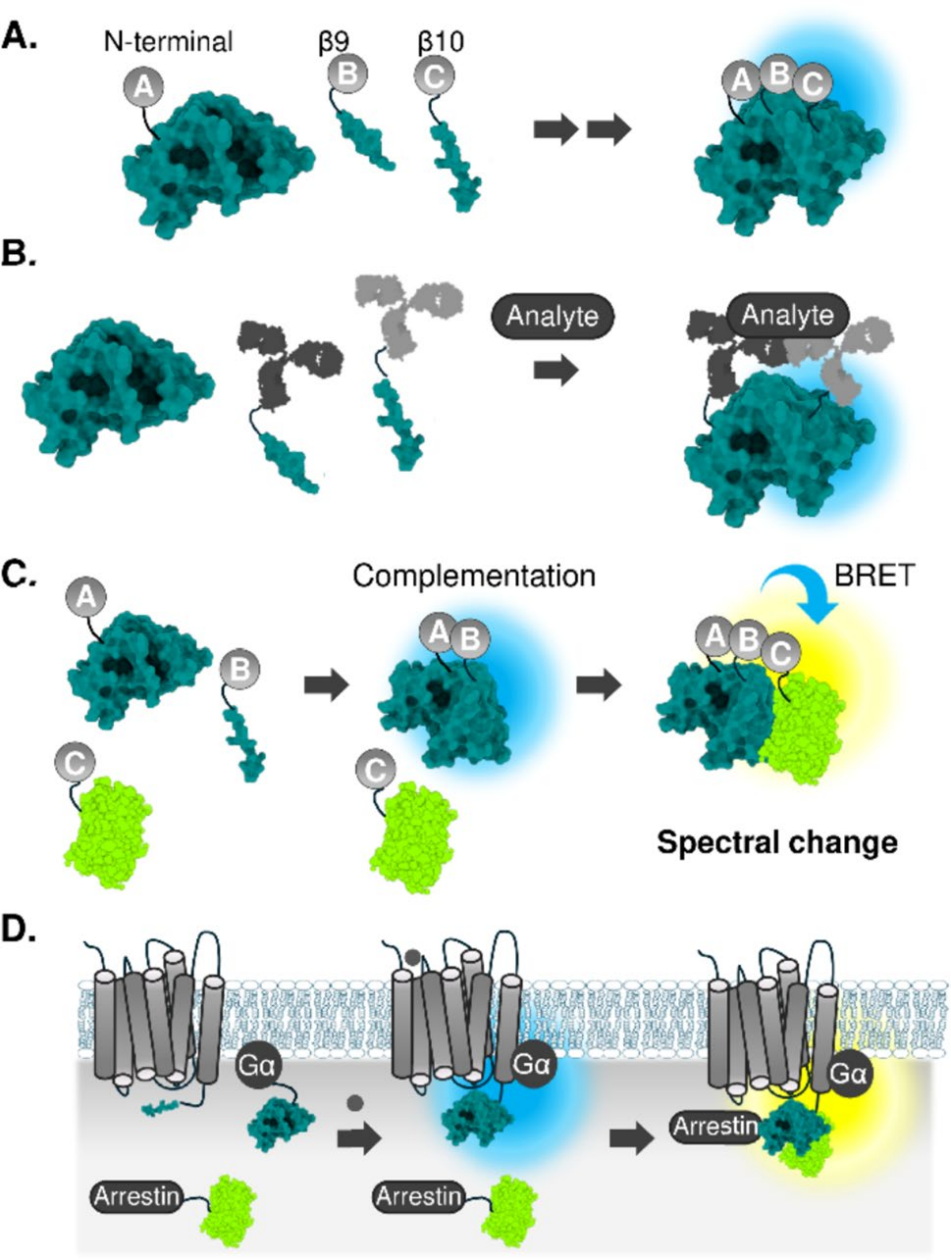
Biosensors to detect ternary interactions. **A** Principle of tri-molecular NanoLuc complementation. **B** Application of ternary complementation to detect antigen. **C** Principle of complementation-dependent BRET for detecting ternary protein complex. **D** Sequential detection of GPCR, G-protein, and arrestin interactions

Another recent advancement in luciferase complementation assays was the discovery of tripartite split NanoLuc fragments. Ternary NanoLuc complementation system was generated by splitting NanoLuc, a 10-strand β-barrel luciferase, into two small C-terminal strands β9 and β10 (11-mers, respectively) and a larger N-terminal body of the protein (Fig. 3A) [38, 39]. In practice, two small fragments are fused to the protein of interest, and an intact larger N-terminal fragment was introduced to the assay system for spontaneous complementation with the two small C-terminal fragments to drive bioluminescence signal emission. This ternary complementation system offers advantages over binary complementation assays in terms of low background signal, and lower possibility of encountering unexpected detection of PPIs owing to the small size of the fragment that would not interfere with the original function of the fused target protein [40].

The method has successfully been applied to the immuno-detection of various analytes, including IL-6 [41], TNFα [42], and immunoglobulin G (IgG) antibodies against SARS-CoV2. The assay system is similar to the enzyme-linked immunosorbent assays (ELISA), where two C-terminal strands fused-antibodies that bind different epitopes on the target analyte are brought into close proximity in the existence of the analyte to emit bioluminescence (Fig. 3B). Recently, robustness of the tripartite fragment complementation were improved by split intein mediated reconstitution of the two small fragments upon interaction [43]. Accordingly, the system shows a good correlation with the result of ELISA, suggesting that the assay system could be an alternative to the ELISA system, with a simple readout of the bioluminescence signal.

### Tripartite interaction detection using split-luciferase complementation and BRET

An alternative approach to decipher ternary protein interactions combines the luciferase complementation assays with bioluminescence resonance energy transfer (BRET) (Fig. 3C). BRET relies on energy transfer from a bioluminescent donor protein to a fluorescent acceptor protein. The efficiency of this energy transfer is dependent on the proximity of the donor and acceptor, typically within 10 nm [44, 45], making BRET a highly sensitive tool for detecting protein–protein interactions. Marine luciferases, with their strong emissions in the 460–490 nm range, are well-suited as BRET donors. Since the development of the initial RLuc-EYFP BRET pair [46], numerous more efficient and spectrally separated BRET systems have been developed [47, 48]. Despite the reduced luminescence of split luciferases compared to full-length enzymes (approximately 0.5–10%), the signal remains sufficient to act as a BRET donor.

By combining luciferase complementation and BRET, it is possible to detect sequential protein interactions. The BRET signal is directly dependent on the complementation of the split luciferase, making this system ideal for studying complex protein networks. A prominent example of this approach is its application in GPCR research, where it was first demonstrated with RLuc and Venus BRET system. In this system, split fragments of the RLuc were fused to dopamine receptors and Venus to G-protein to observe the dimerization of dopamine receptors and their association with G-protein [49]. Since then, this technique enabled the detection of interactions between GPCRs, G-proteins, arrestins, and other GPCR-related effectors such as ERK kinase [50] or GRK (Fig. 3D) [51]. In these assays interactions among GPCR signaling components were simultaneously quantified in real-time. Because the BRET acceptor fluorescent dye can be incorporated into small molecules such as ligands, this approach is also applicable to monitor ligand-GPCR interaction dynamics. For instance, the spontaneous complementation of HiBiT-tagged GPCRs was employed to detect GPCR internalization, while simultaneous BRET measurements between GPCR and fluorescently labeled ligands allowed for the analysis of their pharmacological kinetics [52].

## Design strategies for complementation-based single-chain probes

### Intramolecular complementary probes

**Fig. 4:**
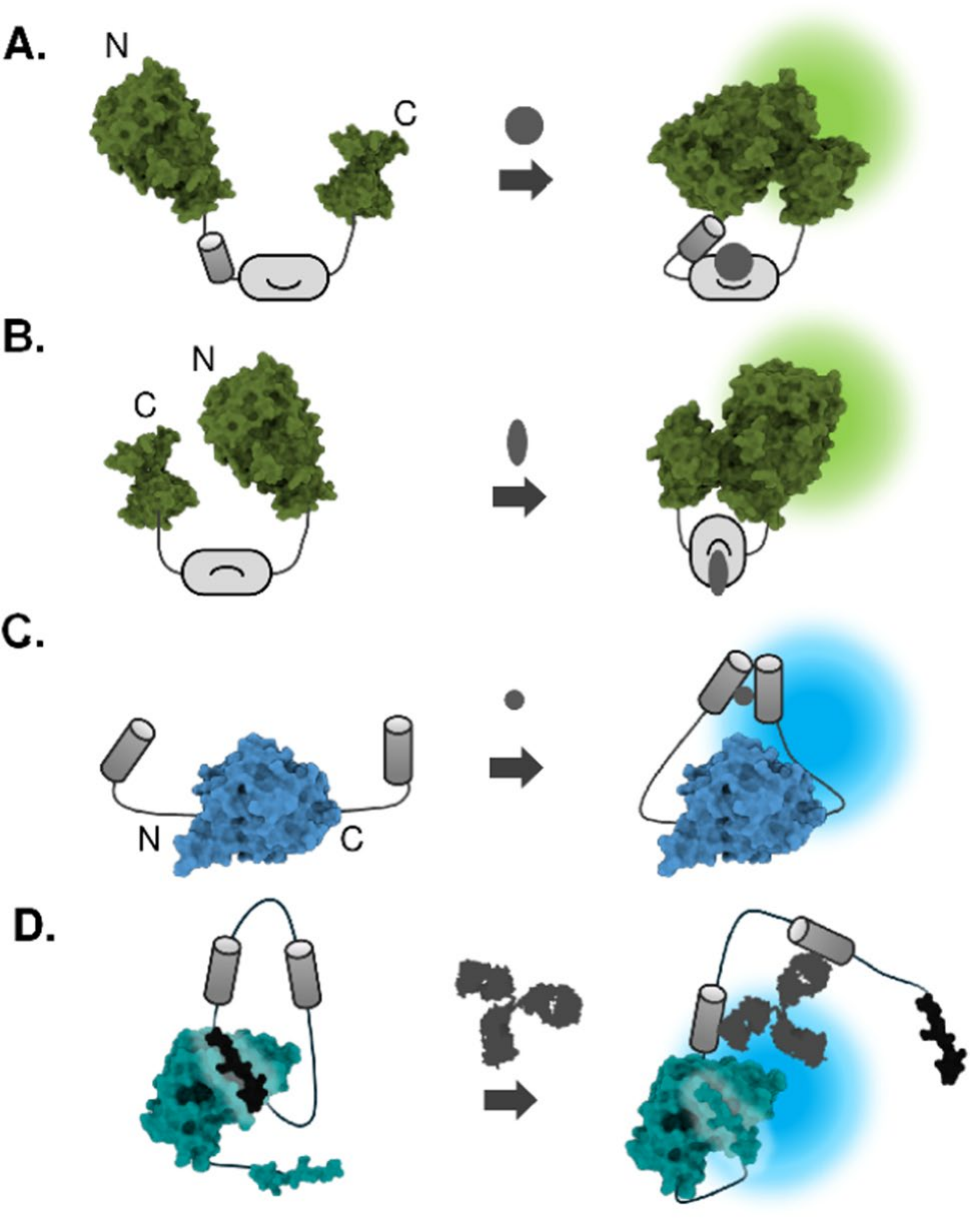
Biosensors based on intramolecular luciferase complementation. **A-C** Intramolecular complementation-based sensor based on different configurations of sensor domain, and N-and C-terminal fragments. **D** Strategy to suppress background signal by engaging catalytic inactive fragment

In contrast to bi- or tri-molecular complementation assays, single-chain probes offer several advantages, such as rapid response times (~ 1 min) and reduced reliance on precise stoichiometric expression. These probes typically consist of split-luciferase fragments connected by a linker region including a sensing domain, enabling intramolecular complementation upon specific molecular events. Early research focused on inserting ligand-binding domains between luciferase fragments to detect ligand-induced conformational changes. For instance, the ligand-binding domains (LBDs) of androgen receptor (AR) or estrogen receptor (ER) have been incorporated into various luciferase types, including *Gaussia* [53], Firefly [54], and Click beetle luciferases [55], to create sensitive hormonal ligand sensors (Fig. 4A). Intramolecular probe was also adopted to detect kinase activity, where the conformational change of the sensor was induced by intramolecular interaction between FHA2 domain that binds phosphorylated amino acid residues by a kinase of Akt [56]. A similar approach has led to the successful detection of other kinases such as ERK [57], and also small compounds, such as Ca^2+^ [58], IP3 [59], and glucose [60].

Above mentioned sensors have the modality of sensing domain fused between the N-terminal and C-terminal fragments of the luciferase. Another type of modality is to use circular permutation, where the locations of the original C-terminal fragment and N-terminal fragment are reversed. A notable example is a cAMP sensor based on circularly permutated firefly luciferase fused to the RIIβB cAMP-binding domain [61]. Circular permutation exploits structural insights into the hinge region, which, can alter luciferase activity when modulated by conformational changes (Fig. 4B) [62]. A circular permutation approach could also be applied to induce a steric hindrance of luciferase activity. In this probe design, circularly permutated RLuc fragments were connected with the linker composing caspase cleavage sequence, which enables the probe to recover its original structure upon cleavage of the linker to emit luminescence [63]. Strictly not a complementation system, modality with a target protein fused to both of the terminal ends of the full-length luciferase was developed as a tension sensor that switches on and off upon mechanical stress on the luciferase induced by fused target proteins (Fig. 4C) [64].

Intramolecular interactions are generally stronger than intermolecular interactions, thus single-chain probes often suffer from high background luminescence possibly resulting from a tendency to undergo spontaneous complementation. Therefore, a careful design is required to ensure optimal distance and orientation between the N- and C-terminal fragments before and after conformational changes. One of the interesting strategies to enhance the sensitivity of the single-chained sensor is to use catalytic inactive fragment. It was demonstrated that the background signal can be lowered by using the NanoLuc C-terminal fragment named “DarkBiT”, which complements the N-terminal LgBiT fragment but without catalytic activity (Fig. 4D) [65]. These studies highlight a promising approach for the development of a single-domain luciferase sensor with a switch based on a conformational change induced by target proteins.

### Protein splicing-mediated protein circulation

Reconstituting luciferase fragments through intein-mediated protein splicing offers an alternative strategy to luciferase complementation assays. The concept of intein-mediated reconstitution of split luciferase fragments emerged from early studies on luciferase complementation assays [5]. In this technique, a protein ligase, named inteins, catalyzes a spontaneous splicing reaction, forming a peptide bond between two protein fragments fused with exteins. Thus, the terminology for splicing-mediated complementation is called “reconstitution”. The reconstitution probe is designed for fusion proteins of split-inteins and split-luciferases, enabling luciferase fragment reconstitution upon proximity of the split fragments. Intramolecular reconstitution of the firefly luciferase was also conducted with another form of protein ligation using the SPY-catcher/SPY-tag system to enhance the thermal stability of the luciferase [66], highlighting the potential of the reconstitution method to engineer luciferase-based biosensors.

**Fig. 5:**
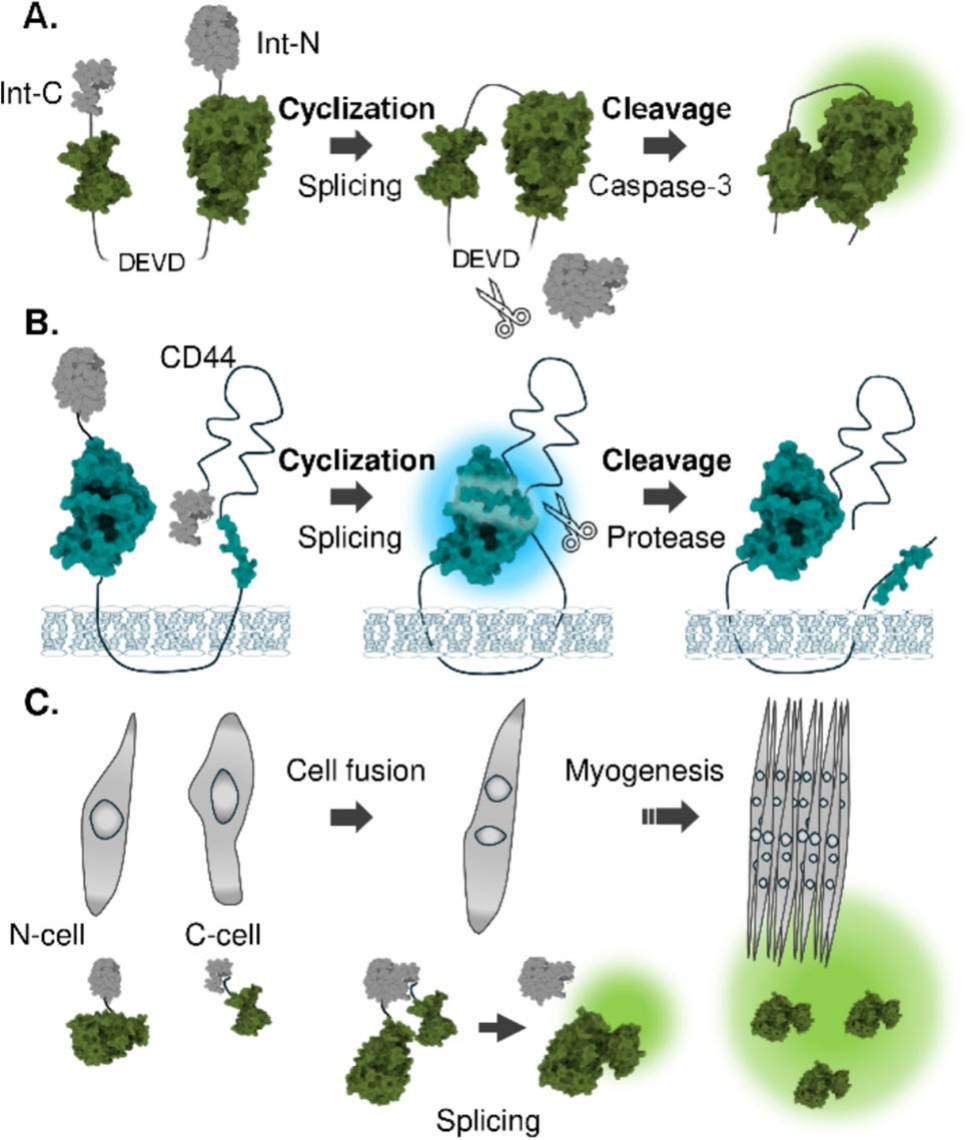
Biosensors based on protein splicing. **A**,** B** Cyclization of Firefly or Nano-luciferase to detect protease activity. **C** Splicing-based reconstitution of FLuc to detect cell fusion progression during myogenesis

While initial studies focused on exploiting protein splicing for efficient reconstitution of the split fragments, subsequent studies have leveraged the covalent, irreversible nature of the system to develop probes with unique functionalities. One strategy of protein splicing research involves protein cyclization, where reconstitution occurs intramolecularly to form a cyclic protein [67]. The cyclization of luciferase for bioluminescent probing was first demonstrated in the detection of caspase-3 activity [68]. Firefly luciferase, engineered with a caspase-3 cleavage sequence (DEVD), was cyclized via protein splicing. Cleavage of the DEVD sequence by caspase-3 relieves structural constraints, leading to an increase in bioluminescence signal (Fig. 5A). The strategy was also applied to detect hepatitis A virus 3C protease activity [69], further demonstrating the versatility of cyclic luciferase to detect protease activity. More recently, luciferase cyclization has been applied to the detection of CD44 ectodomain cleavage [70]. The CD44 ectodomain was conjugated with N- and C-terminal fragments of NanoLuc and cyclized via protein splicing. Cleavage of the cyclized protein by a membrane-associated metalloprotease decreases its signal, enabling the analysis of the role of CD44 in cancer cell migration (Fig. 5B) [70].

Another application of the irreversible nature of protein splicing is to detect biological events such as dissociation of membrane compartmentalization or fusion events between cells. This application takes advantage of the spontaneous reconstitution of the luciferase fragments upon the existence of the two fragments. The combination of split inteins and split FLuc fragments enabled the detection of cell fusion events of the myoblast, where the split half of the fragments are expressed in each cell respectively. The cell fusion events brought a mixture existence of the fragments in a single compartment, enabling the detection of the bioluminescence signal from the reconstituted luciferase (Fig. 5C) [71]. Building upon this elegant concept, one study replaced the FLuc fragments with LgBiT and SmBiT, smaller and more sensitive fragments [72], and another system used the HiBiT system, where the HiBiT and body of the NanoLuc are used instead of fusion probes consisting of split inteins and split FLuc [73]. The approach is versatile because it is not limited to cell fusion but can be applied to a variety of membrane fusion processes. This opens possibilities for future research to develop a biosensor for various types of biological fusion events.

## Expanding applications of split-luciferase complementation assay beyond PPI detection

### Multicolor luciferase complementation system

Beetle luciferases share a conserved protein structure, enabling N-terminal fragments from one species to complement with C-terminal fragments from another. Owing to this character, multicolor emission from complemented fragments was demonstrated with click beetle green luciferase (CBG) and click beetle red luciferase (CBR) developed from Caribbean click beetle *Pyrophorus plagiophthalamus* [74], which share 99% amino acid identity despite distinct emission wavelengths of green (Em. max 540 nm) and red (Em. max 615 nm) [17]. By fusing the same C-terminal fragment with either CBG or CBR N-terminal fragments, a sensor capable of detecting both agonist- and antagonist-mediated conformational changes within a single molecule has been developed [75].

**Fig. 6:**
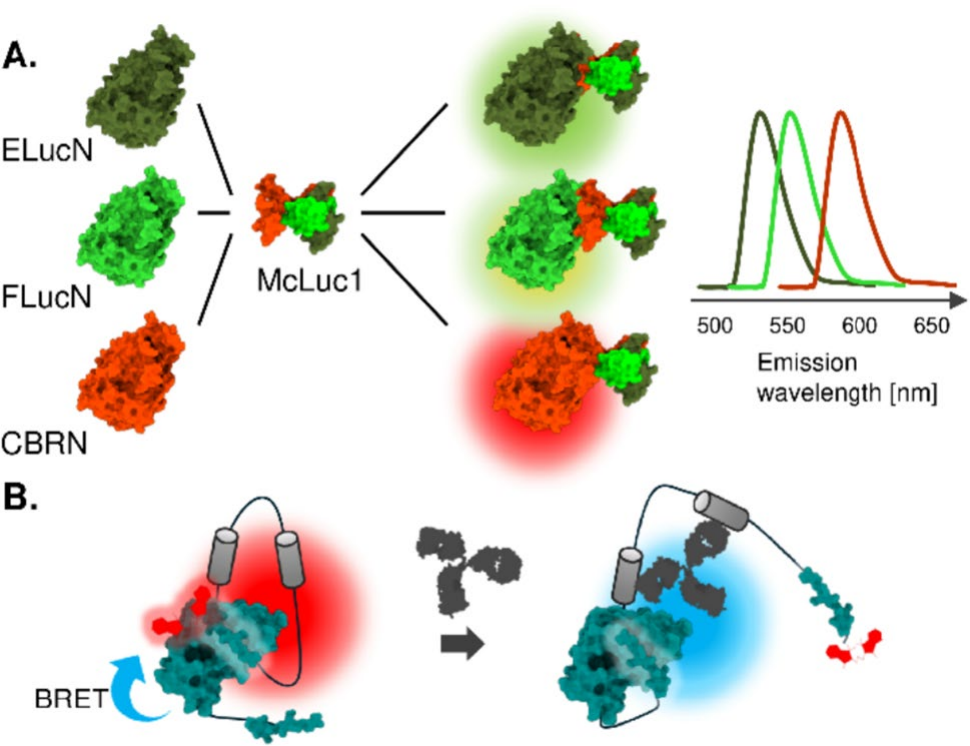
Schematic illustration of the multicolor bioluminescent sensors. **A** Spectral separation of bioluminescence signal from complemented luciferase with different N- and C- terminal fragment pairs. **B** Multicolor emission achieved through complementation of native and fluorescent dye-conjugated fragments, using BRET to induce a red-shift

To further expand the palette of emission colors, a multi-complementary C-terminal fragment (McLuc1) was engineered through random mutagenesis of the CBR C-terminal fragment. McLuc1 can complement various types of N-terminal fragments of beetle luciferases, enabling simultaneous detection of multiple PPIs by spectral unmixing (Fig. 6A) [16]. This approach has been successfully applied to monitor Smad1-Smad4 and Smad2-Smad4 interactions during *Xenopus laevis* development, demonstrating the potential for multicolor PPI detection in vivo [16]. Additionally, McLuc1 has been used for ratiometric cAMP sensor and their application to detect changes in cAMP levels in live mice, further validating the versatility of this technology [76].

For marine luciferase, the color tuning for multi-color detection was accomplished by using BRET to red-shift the emission wavelength of the bioluminescence signal. For example, the NanoLuc N-terminal fragment can complement either the native SmBiT or the fluorescent dye-adjacent SmBiT, but the light produced by the latter will exhibit a red shift as a result of BRET occurring between the complemented NanoLuc fragment and the fluorescent dye (Fig. 6B). Ratiometric sensor based on this method developed by utilizing a conformational change of the sensor that allows a switch of complementation partner of LgBiT upon antibody binding to the antigen within the sensor [77, 78]. A similar approach was used to construct a ratio metric sensor for Zn^2+^ ion using a conformational change induced by the binding of zinc ion to the Zn^2+^-binding domains [79]. In another example, fluorescent proteins were adopted as BRET acceptors, and affinity-tuned multicolor bioluminescent Ca^2+^ indicators have enabled the visualization of local Ca^2+^ dynamics in living cells [80]. While the emission spectra of luciferases tend to be broader compared to fluorescent proteins, the elegant approach of ratiometric detection offers advantages in calibrating the detected signal. This provides a unique and powerful strategy for developing luciferase complementation sensors with robust and reliable readouts.

### Detection of nucleic acids using nucleotide-binding proteins

Complementation of luciferase fragments can also be driven by mere proximity. In this strategy, luciferase activity is recovered by complementation of the split fragments being adjacent to each other. This approach is effective especially for detecting nucleic acids, by bringing luciferase fragments into adjacent positions by harnessing nucleotide-binding proteins which can be designed to bind to the desired nucleotide sequences. Early studies harnessed arginine-rich motif peptides, well-characterized RNA-binding peptides that undergo conformational changes upon binding to specific RNAs. These peptides were inserted into intramolecularly split RLuc fragments [81]. By exchanging the nucleotide-binding domain to methyl-CpG binding domains, CpG methylated status was quantified [82]. Similarly, fusions of MS2 coat protein (MCP) and PP7 coat protein (PCP) with NanoLuc or FLuc fragments were used to quantify the level of the bait RNA [83].

**Fig. 7:**
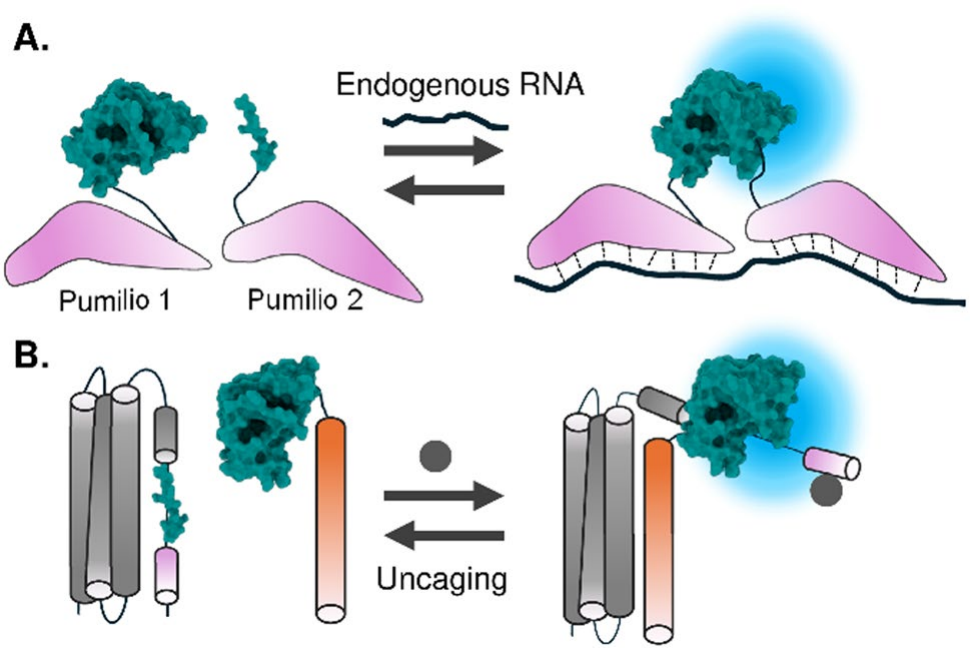
**A** The detection method of endogenous RNA in a living cell using proximity-dependent complementation of luciferase. **B** Detection of a target binder through a switch in complementation induced by steric hindrance

One of the challenges in cell biology is tracking and quantifying RNA levels within living cells. To address this, RNA-binding protein pumilio has been utilized as an RNA-binding domain to detect a specific target RNA. The initial attempt demonstrated that by combining luciferase fragments with pumilio, it was possible to detect user-defined single-stranded RNA through proximity-based interactions in vitro [84]. More recently, to fully exploit the potential of pumilio, the system was adapted to detect endogenous β-actin mRNA in living cells (Fig. 7A) [85]. In this method, a NanoLuc fragment fused to pumilio was designed to bind to two adjacent sequences within the β-actin mRNA, which successfully quantified the spatiotemporal dynamics of the β-actin RNA in both living cells and cultured neurons [85]. Because the method is generalizable to detect various RNA by modifying RNA recognition repeats of pumilio, the technique has opened avenues for applications ranging from subcellular to in vivo imaging.

### Steric hinderance-dependent luciferase complementation

Another strategy to switch the reassembly of the split luciferase fragments is to introduce a steric barrier to prevent the association of the fragments. This strategy was applied to a type of sensor that involves uncaging the small NanoLuc C-fragment (SmBiT). In this strategy, SmBiT is positioned within a sensing module of a sensor, where its interaction with LgBiT is blocked in the basal state. Upon binding to a specific analyte, the sensing module undergoes a conformational change, exposing SmBiT and enabling its complementation with LgBiT, resulting in a bioluminescent signal. A notable example of this approach is de novo designed the “lucCage” system (Fig. 7B). In this system, the steric hinderance is released when the sensor's binding domain interacts with the target analyte [86]. The methodology successfully detected the target protein using a single-domain antibody [86] or antigen [87] as a binding domain within the sensing module.

In another application, SmBiT was caged within the C-terminal loop of titin, a protein responsible for muscle contraction. This design allowed for the development of a tension sensor because the SmBiT is uncaged when tension is applied to the sensor [88]. These strategies demonstrate the utility of the small size of the SmBiT tag to be used for caging and provide a versatile design strategy for these uncaging-based bioluminescence sensors.

## Future perspectives

Over the past two decades, luciferase complementation techniques have emerged as a powerful tool with broad applications in biology. The development of NanoLuc complementation assays, with their small protein size, brightness, and tunable affinity, has opened new possibilities for research. These technological advancements have led to the development of a diverse array of luciferase-based biosensors for some fields in cellular biology, including cell death and GPCR signaling, which could be beneficial for investigating biological events within specific cellular contexts [89, 90]. While the community has made significant advancements, the full potential of luciferase complementation-based biosensors is yet to be fully realized, promising even more sophisticated designs and applications in the future.

One of the most promising applications of split luciferase complementation is high-throughput screening for identifying molecules that modulate biological functions [91]. It has been shown that the method is applicable to the scale of screening using 1536-well plates, which meets the requirement of high throughput screening [92]. Despite the many advantages of luciferase-based assays, there are still some current limitations. Steric hindrance and direct effects on luciferase activity can sometimes hinder the detection of certain compounds [93]. Additionally, there’s still room for improvement in the robustness of cell-free assay systems, such as the stability of the fragmented luciferase in vitro. To overcome these challenges, we need to adopt a multi-faceted approach and develop innovative strategies for quality control.

Another high-potential application of luciferase complementation is in vivo imaging. While bioluminescence offers a non-invasive approach to visualize biological processes, the visible light emitted by both beetle and marine luciferases is difficult to penetrate deep tissues. To address this limitation, researchers have explored various strategies to red-shift the emission wavelength, including genetic engineering and coevolution of luciferase and its substrate [94–96], red-shifted substrates [97, 98] and use of BRET [99, 100]. However, the lower bioluminescence signal of complemented luciferases compared to full-length enzymes remains a challenge that requires further investigation. By continuing to innovate and explore new applications, luciferase complementation techniques would continue to contribute to biological research and pharmacological applications.

## Data Availability

The manuscript has no associated data.

## References

[1] N. Johnsson, A. Varshavsky, Proc. Natl. Acad. Sci. **91**, 10340–10344 (1994). 10.1073/pnas.91.22.10340 7937952 10.1073/pnas.91.22.10340 PMC45015

[2] I. Ghosh, A.D. Hamilton, L. Regan, J. Am. Chem. Soc. **122**, 5658–5659 (2000). 10.1021/ja994421w

[3] J.N. Pelletier, F.-X. Campbell-Valois, S.W. Michnick, Proc. Natl. Acad. Sci. **95**, 12141–12146 (1998). 10.1073/pnas.95.21.12141 9770453 10.1073/pnas.95.21.12141 PMC22798

[4] A. Galarneau, M. Primeau, L.-E. Trudeau, S.W. Michnick, Nat. Biotechnol. **20**, 619–622 (2002). 10.1038/nbt0602-619 12042868 10.1038/nbt0602-619

[5] T. Ozawa, A. Kaihara, M. Sato, K. Tachihara, Y. Umezawa, Anal. Chem. **73**, 2516–2521 (2001). 10.1021/ac0013296 11403293 10.1021/ac0013296

[6] R. Paulmurugan, Y. Umezawa, S.S. Gambhir, Proc. Natl. Acad. Sci. **99**, 15608–15613 (2002). 10.1073/pnas.242594299 12438689 10.1073/pnas.242594299 PMC137764

[7] S.B. Kim, T. Furuta, Front. Chem. Biol. **3**, (2024). 10.3389/fchbi.2024.1459397

[8] Y. Ohmuro-Matsuyama, C.-I. Chung, H. Ueda, BMC Biotechnol. **13**, 31 (2013). 10.1186/1472-6750-13-31 23536995 10.1186/1472-6750-13-31 PMC3626928

[9] S.H.D. Haddock, M.A. Moline, J.F. Case, Annu. Rev. Mar. Sci. **2**, 443–493 (2010). 10.1146/annurev-marine-120308-081028 10.1146/annurev-marine-120308-081028 21141672

[10] G. Kawamura, M. Hattori, K. Takamatsu, T. Tsukada, Y. Ninomiya, I. Benjamin, P. Sassone-Corsi, T. Ozawa, T. Tamaru, Commun. Biol. **1**, 204 (2018). 10.1038/s42003-018-0209-1 30480104 10.1038/s42003-018-0209-1 PMC6250677

[11] G.F. Pelentir, V.R. Bevilaqua, V.R. Viviani, Photochem. Photobiol. Sci. **18**, 2061–2070 (2019). 10.1039/c9pp00174c 31339127 10.1039/c9pp00174c

[12] V.R. Viviani, E.J.H. Bechara, Y. Ohmiya, Biochemistry **38**, 8271–8279 (1999). 10.1021/bi9900830 10387072 10.1021/bi9900830

[13] C. Carrasco-López, N.M. Lui, S. Schramm, P. Naumov, Nat. Rev. Chem. **5**, 4–20 (2021). 10.1038/s41570-020-00238-1 37118106 10.1038/s41570-020-00238-1

[14] K.E. Luker, M.C.P. Smith, G.D. Luker, S.T. Gammon, H. Piwnica-Worms, D. Piwnica-Worms, Proc. Natl. Acad. Sci. **101**, 12288–12293 (2004). 10.1073/pnas.0404041101 15284440 10.1073/pnas.0404041101 PMC514471

[15] R. Paulmurugan, S.S. Gambhir, Anal. Chem. **79**, 2346–2353 (2007). 10.1021/ac062053q 17295448 10.1021/ac062053q PMC3198827

[16] N. Hida, M. Awais, M. Takeuchi, N. Ueno, M. Tashiro, C. Takagi, T. Singh, M. Hayashi, Y. Ohmiya, T. Ozawa, PLoS ONE **4**, e5868 (2009). 10.1371/journal.pone.0005868 19536355 10.1371/journal.pone.0005868 PMC2697115

[17] V. Villalobos, S. Naik, M. Bruinsma, R.S. Dothager, M.-H. Pan, M. Samrakandi, B. Moss, A. Elhammali, D. Piwnica-Worms, Chem. Biol. **17**, 1018–1029 (2010). 10.1016/j.chembiol.2010.06.018 20851351 10.1016/j.chembiol.2010.06.018 PMC2943495

[18] M. Chen, C. Yan, F. Qin, X.-E. Zhang, Anal. Chem. **94**, 13700–13709 (2022). 10.1021/acs.analchem.2c01238 36135776 10.1021/acs.analchem.2c01238

[19] M.P. Hall, J. Unch, B.F. Binkowski, M.P. Valley, B.L. Butler, M.G. Wood, P. Otto, K. Zimmerman, G. Vidugiris, T. Machleidt, M.B. Robers, H.A. Benink, C.T. Eggers, M.R. Slater, P.L. Meisenheimer, D.H. Klaubert, F. Fan, L.P. Encell, K.V. Wood, ACS Chem. Biol. **7**, 1848–1857 (2012). 10.1021/cb3002478 22894855 10.1021/cb3002478 PMC3501149

[20] D.S. Auld, J. Narahari, P. Ho, D. Casalena, V. Nguyen, E. Cirbaite, D. Hughes, J. Daly, B. Webb, Biochemistry **57**, 4700–4706 (2018). 10.1021/acs.biochem.8b00290 29641191 10.1021/acs.biochem.8b00290

[21] S.B. Kim, M. Torimura, H. Tao, Bioconjug. Chem. **24**, 2067–2075 (2013). 10.1021/bc400411h 24237362 10.1021/bc400411h

[22] Y. Ohmuro-Matsuyama, T. Furuta, H. Matsui, M. Kanai, H. Ueda, ACS Chem. Biol. **17**, 864–872 (2022). 10.1021/acschembio.1c00897 35293729 10.1021/acschembio.1c00897

[23] A. Kaihara, Y. Umezawa, Chem. Asian J. **3**, 38–45 (2008). 10.1002/asia.200700186 18058892 10.1002/asia.200700186

[24] E. Stefan, S. Aquin, N. Berger, C.R. Landry, B. Nyfeler, M. Bouvier, S.W. Michnick, Proc. Natl. Acad. Sci. **104**, 16916–16921 (2007). 10.1073/pnas.0704257104 17942691 10.1073/pnas.0704257104 PMC2040481

[25] R. Paulmurugan, S.S. Gambhir, Anal. Chem. **75**, 1584–1589 (2003). 10.1021/ac020731c 12705589 10.1021/ac020731c PMC4154785

[26] I. Remy, S.W. Michnick, Nat. Methods **3**, 977–979 (2006). 10.1038/nmeth979 17099704 10.1038/nmeth979

[27] S.B. Kim, Protein Eng. Des. Sel. **25**, 261–269 (2012). 10.1093/protein/gzs016 22514115 10.1093/protein/gzs016

[28] O. Dagliyan, A. Krokhotin, I. Ozkan-Dagliyan, A. Deiters, C.J. Der, K.M. Hahn, N.V. Dokholyan, Nat. Commun. **9**, 4042 (2018). 10.1038/s41467-018-06531-4 30279442 10.1038/s41467-018-06531-4 PMC6168510

[29] T. Azad, A. Tashakor, S. Hosseinkhani, Anal. Bioanal. Chem. **406**, 5541–5560 (2014). 10.1007/s00216-014-7980-8 25002334 10.1007/s00216-014-7980-8

[30] S.-B. Kim, R. Paulmurugan, Anal. Sci. **37**, 233–247 (2021). 10.2116/analsci.20R003 32963202 10.2116/analsci.20R003

[31] M.K. Schwinn, T. Machleidt, K. Zimmerman, C.T. Eggers, A.S. Dixon, R. Hurst, M.P. Hall, L.P. Encell, B.F. Binkowski, K.V. Wood, ACS Chem. Biol. **13**, 467–474 (2018). 10.1021/acschembio.7b00549 28892606 10.1021/acschembio.7b00549

[32] D.C. Ranawakage, T. Takada, Y. Kamachi, Sci. Rep. **9**, 6895 (2019). 10.1038/s41598-019-43319-y 31053795 10.1038/s41598-019-43319-y PMC6499798

[33] C.W. White, L.E. Kilpatrick, K.D.G. Pfleger, S.J. Hill, iScience **24**, 102011 (2021). 10.1016/j.isci.2020.102011 33490919 10.1016/j.isci.2020.102011 PMC7809502

[34] H. Lin, K. Riching, M.P. Lai, D. Lu, R. Cheng, X. Qi, J. Wang, ACS Med. Chem. Lett. **15**, 1367–1375 (2024). 10.1021/acsmedchemlett.4c00271 39140070 10.1021/acsmedchemlett.4c00271 PMC11318018

[35] M.K. Schwinn, L.S. Steffen, K. Zimmerman, K.V. Wood, T. Machleidt, Sci. Rep. **10**, 8953 (2020). 10.1038/s41598-020-65832-1 32488146 10.1038/s41598-020-65832-1 PMC7265437

[36] M. Soave, B. Kellam, J. Woolard, S.J. Briddon, S.J. Hill, SLAS Discov. **25**, 186–194 (2020). 10.1177/2472555219880475 31583945 10.1177/2472555219880475 PMC6974774

[37] M. Endo, M. Miyasaki, Q. Li, G. Kawamura, T. Ozawa, Anal. Sci. **35**, 835–838 (2019). 10.2116/analsci.19C003 31281129 10.2116/analsci.19C003

[38] A.S. Dixon, S.J. Kim, B.K. Baumgartner, S. Krippner, S.C. Owen, Sci. Rep. **7**, 8186 (2017). 10.1038/s41598-017-07569-y 28811487 10.1038/s41598-017-07569-y PMC5557857

[39] Y. Ohmuro-Matsuyama, H. Ueda, Anal. Chem. **90**, 3001–3004 (2018). 10.1021/acs.analchem.7b05140 29446920 10.1021/acs.analchem.7b05140

[40] M. Oliayi, R. Emamzadeh, M. Rastegar, M. Nazari, Anal. Methods **15**, 3924–3931 (2023). 10.1039/D3AY00512G 37545367 10.1039/d3ay00512g

[41] M.P. Hall, V.A. Kincaid, E.A. Jost, T.P. Smith, R. Hurst, S.K. Forsyth, C. Fitzgerald, V.T. Ressler, K. Zimmermann, D. Lazar, M.G. Wood, K.V. Wood, T.A. Kirkland, L.P. Encell, T. Machleidt, M.L. Dart, Anal. Chem. **93**, 5177–5184 (2021). 10.1021/acs.analchem.0c05074 33730483 10.1021/acs.analchem.0c05074

[42] S.J. Kim, A.S. Dixon, P.C. Adamovich, P.D. Robinson, S.C. Owen, ACS Sens. **6**, 1807–1814 (2021). 10.1021/acssensors.0c02642 34010570 10.1021/acssensors.0c02642

[43] Z. Yao, J. Kim, B. Geng, J. Chen, V. Wong, A. Lyakisheva, J. Snider, M.R. Dimlić, S. Raić, I. Stagljar, Mol. Syst. Biol. 1–19 (2024). 10.1038/s44320-024-00081-2 10.1038/s44320-024-00081-2 PMC11791039 39668253

[44] H. Dacres, J. Wang, M.M. Dumancic, S.C. Trowell, Anal. Chem. **82**, 432–435 (2010). 10.1021/ac9022956 19957970 10.1021/ac9022956

[45] F. Weihs, J. Wang, K.D.G. Pfleger, H. Dacres, Anal. Chim. Acta X **6**, 100059 (2020). 10.1016/j.acax.2020.100059 33392495 10.1016/j.acax.2020.100059 PMC7772631

[46] Y. Xu, D.W. Piston, C.H. Johnson, Proc. Natl. Acad. Sci. **96**, 151–156 (1999). 10.1073/pnas.96.1.151 9874787 10.1073/pnas.96.1.151 PMC15108

[47] K. Saito, Y.-F. Chang, K. Horikawa, N. Hatsugai, Y. Higuchi, M. Hashida, Y. Yoshida, T. Matsuda, Y. Arai, T. Nagai, Nat. Commun. **3**, 1262 (2012). 10.1038/ncomms2248 23232392 10.1038/ncomms2248 PMC3535334

[48] A. Takai, M. Nakano, K. Saito, R. Haruno, T.M. Watanabe, T. Ohyanagi, T. Jin, Y. Okada, T. Nagai, Proc. Natl. Acad. Sci. **112**, 4352–4356 (2015). 10.1073/pnas.1418468112 25831507 10.1073/pnas.1418468112 PMC4394297

[49] E. Urizar, H. Yano, R. Kolster, C. Galés, N. Lambert, J.A. Javitch, Nat. Chem. Biol. **7**, 624–630 (2011). 10.1038/nchembio.623 21785426 10.1038/nchembio.623 PMC3158273

[50] J.S. Smith, T.F. Pack, A. Inoue, C. Lee, K. Zheng, I. Choi, D.S. Eiger, A. Warman, X. Xiong, Z. Ma, G. Viswanathan, I.M. Levitan, L.K. Rochelle, D.P. Staus, J.C. Snyder, A.W. Kahsai, M.G. Caron, S. Rajagopal, Science **371**, (2021). 10.1126/science.aay1833 10.1126/science.aay1833 PMC8005335 33479120

[51] K. Kawakami, M. Yanagawa, S. Hiratsuka, M. Yoshida, Y. Ono, M. Hiroshima, M. Ueda, J. Aoki, Y. Sako, A. Inoue, Nat. Commun. **13**, 487 (2022). 10.1038/s41467-022-28056-7 35078997 10.1038/s41467-022-28056-7 PMC8789823

[52] M.E. Boursier, S. Levin, K. Zimmerman, T. Machleidt, R. Hurst, B.L. Butler, C.T. Eggers, T.A. Kirkland, K.V. Wood, R. Friedman Ohana, J. Biol. Chem. **295**, 5124–5135 (2020). 10.1074/jbc.RA119.011952 32107310 10.1074/jbc.RA119.011952 PMC7152755

[53] S.B. Kim, M. Sato, H. Tao, Anal. Chem. **81**, 67–74 (2009). 10.1021/ac801658y 19061336 10.1021/ac801658y

[54] S.B. Kim, M. Awais, M. Sato, Y. Umezawa, H. Tao, Anal. Chem. **79**, 1874–1880 (2007). 10.1021/ac061934u 17269793 10.1021/ac061934u

[55] O. Takenouchi, A. Kanno, H. Takakura, M. Hattori, T. Ozawa, Bioconjug. Chem. **27**, 2689–2694 (2016). 10.1021/acs.bioconjchem.6b00466 27690388 10.1021/acs.bioconjchem.6b00466

[56] L. Zhang, K.C. Lee, M.S. Bhojani, A.P. Khan, A. Shilman, E.C. Holland, B.D. Ross, A. Rehemtulla, Nat. Med. **13**, 1114–1119 (2007). 10.1038/nm1608 17694068 10.1038/nm1608

[57] Y. Wu, J.R. Walker, M. Westberg, L. Ning, M. Monje, T.A. Kirkland, M.Z. Lin, Y. Su, ACS Cent. Sci. **9**, 719–732 (2023). 10.1021/acscentsci.3c00074 37122464 10.1021/acscentsci.3c00074 PMC10141594

[58] I. Farhana, M.N. Hossain, K. Suzuki, T. Matsuda, T. Nagai, ACS Sens. **4**, 1825–1834 (2019). 10.1021/acssensors.9b00531 31276380 10.1021/acssensors.9b00531

[59] F. Ataei, M. Torkzadeh-Mahani, S. Hosseinkhani, Biosens. Bioelectron. **41**, 642–648 (2013). 10.1016/j.bios.2012.09.037 23122229 10.1016/j.bios.2012.09.037

[60] K. Teasley Hamorsky, C.M. Ensor, Y. Wei, S. Daunert, Angew. Chem. Int. Ed. **47**, 3718–3721 (2008). 10.1002/anie.200704440 10.1002/anie.200704440 18383457

[61] F. Fan, B.F. Binkowski, B.L. Butler, P.F. Stecha, M.K. Lewis, K.V. Wood, ACS Chem. Biol. **3**, 346–351 (2008). 10.1021/cb8000414 18570354 10.1021/cb8000414

[62] B.F. Binkowski, B.L. Butler, P.F. Stecha, C.T. Eggers, P. Otto, K. Zimmerman, G. Vidugiris, M.G. Wood, L.P. Encell, F. Fan, K.V. Wood, ACS Chem. Biol. **6**, 1193–1197 (2011). 10.1021/cb200248h 21932825 10.1021/cb200248h

[63] R. Mokhtar-Ahmadabadi, Z. Madadi, S. Akbari-Birgani, C. Grillon, L. Hasani, S. Hosseinkhani, S. Zareian, Biochem. Biophys. Res. Commun. **506**, 1032–1039 (2018). 10.1016/j.bbrc.2018.11.009 30409426 10.1016/j.bbrc.2018.11.009

[64] S.B. Kim, R. Nishihara, D. Citterio, K. Suzuki, Bioconjug. Chem. **27**, 354–362 (2016). 10.1021/acs.bioconjchem.5b00421 26322739 10.1021/acs.bioconjchem.5b00421

[65] A. Gräwe, M. Merkx, ACS Sens. **7**, 3800–3808 (2022). 10.1021/acssensors.2c01726 36450135 10.1021/acssensors.2c01726 PMC9791688

[66] M. Si, Q. Xu, L. Jiang, H. Huang, PLoS ONE **11**, e0162318 (2016). 10.1371/journal.pone.0162318 27658030 10.1371/journal.pone.0162318 PMC5033358

[67] T.C. Evans, J. Benner, M.-Q. Xu, J. Biol. Chem. **274**, 18359–18363 (1999). 10.1074/jbc.274.26.18359 10373440 10.1074/jbc.274.26.18359

[68] A. Kanno, Y. Yamanaka, H. Hirano, Y. Umezawa, T. Ozawa, Angew. Chem. Int. Ed. **46**, 7595–7599 (2007). 10.1002/anie.200700538 10.1002/anie.200700538 17722214

[69] J. Zhou, D. Wang, Y. Xi, X. Zhu, Y. Yang, M. Lv, C. Luo, J. Chen, X. Ye, L. Fang, S. Xiao, Biochem. Biophys. Res. Commun. **488**, 621–627 (2017). 10.1016/j.bbrc.2017.05.063 28501618 10.1016/j.bbrc.2017.05.063 PMC7092888

[70] N. Noda, T. Ozawa, J. Cell Sci. **135**, jcs259314 (2022). 10.1242/jcs.259314 35194645 10.1242/jcs.259314

[71] Q. Li, H. Yoshimura, M. Komiya, K. Tajiri, M. Uesugi, Y. Hata, T. Ozawa, Analyst **143**, 3472–3480 (2018). 10.1039/C8AN00285A 29944152 10.1039/c8an00285a

[72] M. Orioka, M. Eguchi, Y. Mizui, Y. Ikeda, A. Sakama, Q. Li, H. Yoshimura, T. Ozawa, D. Citterio, Y. Hiruta, Bioconjug. Chem. **33**, 496–504 (2022). 10.1021/acs.bioconjchem.2c00035 35184558 10.1021/acs.bioconjchem.2c00035

[73] M. Isobe, Y. Suzuki, H. Sugiura, M. Shibata, Y. Ohsaki, S. Kametaka, Biomed. Res. **43**, 107–114 (2022). 10.2220/biomedres.43.107 35989286 10.2220/biomedres.43.107

[74] U. Stolz, S. Velez, K.V. Wood, M. Wood, J.L. Feder, Proc. Natl. Acad. Sci. **100**, 14955–14959 (2003). 10.1073/pnas.2432563100 14623957 10.1073/pnas.2432563100 PMC299859

[75] S.B. Kim, Y. Umezawa, K.A. Kanno, H. Tao, ACS Chem. Biol. **3**, 359–372 (2008). 10.1021/cb800004s 18570355 10.1021/cb800004s

[76] M. Takeuchi, Y. Nagaoka, T. Yamada, H. Takakura, T. Ozawa, Anal. Chem. **82**, 9306–9313 (2010). 10.1021/ac102692u 20979393 10.1021/ac102692u

[77] Y. Ni, R. Arts, M. Merkx, ACS Sens. **4**, 20–25 (2019). 10.1021/acssensors.8b01381 30525479 10.1021/acssensors.8b01381 PMC6350203

[78] E.A. van Aalen, J.J.J. Lurvink, L. Vermeulen, B. van Gerven, Y. Ni, R. Arts, M. Merkx, ACS Sens. **9**, 1401–1409 (2024). 10.1021/acssensors.3c02478 38380622 10.1021/acssensors.3c02478 PMC10964239

[79] C.M.S. Michielsen, E.A. van Aalen, M. Merkx, ACS Chem. Biol. **17**, 1567–1576 (2022). 10.1021/acschembio.2c00227 35611686 10.1021/acschembio.2c00227 PMC9207811

[80] M.N. Hossain, K. Suzuki, M. Iwano, T. Matsuda, T. Nagai, ACS Chem. Biol. **13**, 1862–1871 (2018). 10.1021/acschembio.7b01014 29494125 10.1021/acschembio.7b01014

[81] T. Andou, T. Endoh, M. Mie, E. Kobatake, Anal. Bioanal. Chem. **393**, 661–668 (2009). 10.1007/s00216-008-2473-2 18979090 10.1007/s00216-008-2473-2

[82] A.H. Badran, J.L. Furman, A.S. Ma, T.J. Comi, J.R. Porter, I. Ghosh, Anal. Chem. **83**, 7151–7157 (2011). 10.1021/ac2015239 21797230 10.1021/ac2015239 PMC3206592

[83] L.P. Halbers, K.H. Cole, K.K. Ng, E.B. Fuller, C.E.T. Chan, C. Callicoatte, M. Metcalfe, C.C. Chen, A.A. Barhoosh, E. Reid-McLaughlin, A.D. Kent, Z.R. Torrey, O. Steward, A. Lupták, J.A. Prescher, Nat. Commun. **15**, 9992 (2024). 10.1038/s41467-024-54263-5 39557883 10.1038/s41467-024-54263-5 PMC11574019

[84] J.L. Furman, A.H. Badran, O. Ajulo, J.R. Porter, C.I. Stains, D.J. Segal, I. Ghosh, J. Am. Chem. Soc. **132**, 11692–11701 (2010). 10.1021/ja104395b 20681585 10.1021/ja104395b PMC2954892

[85] M. Eguchi, H. Yoshimura, Y. Ueda, T. Ozawa, ACS Sens. **8**, 4055–4063 (2023). 10.1021/acssensors.3c01080 37889477 10.1021/acssensors.3c01080

[86] A. Quijano-Rubio, H.-W. Yeh, J. Park, H. Lee, R.A. Langan, S.E. Boyken, M.J. Lajoie, L. Cao, C.M. Chow, M.C. Miranda, J. Wi, H.J. Hong, L. Stewart, B.-H. Oh, D. Baker, Nature **591**, 482–487 (2021). 10.1038/s41586-021-03258-z 33503651 10.1038/s41586-021-03258-z PMC8074680

[87] J.Z. Zhang, H.-W. Yeh, A.C. Walls, B.I.M. Wicky, K.R. Sprouse, L.A. VanBlargan, R. Treger, A. Quijano-Rubio, M.N. Pham, J.C. Kraft, I.C. Haydon, W. Yang, M. DeWitt, J.E. Bowen, C.M. Chow, L. Carter, R. Ravichandran, M.H. Wener, L. Stewart, D. Veesler, M.S. Diamond, A.L. Greninger, D.M. Koelle, D. Baker, Nat. Biotechnol. **40**, 1336–1340 (2022). 10.1038/s41587-022-01280-8 35484405 10.1038/s41587-022-01280-8 PMC9463068

[88] B.L. Zhong, J.M. Elliot, P. Wang, H. Li, R.N. Hall, B. Wang, M. Prakash, A.R. Dunn, ACS Sens. **9**, 3489–3495 (2024). 10.1021/acssensors.3c02664 38973210 10.1021/acssensors.3c02664 PMC11839233

[89] S. Hosseinkhani, M. Amandadi, P. Ghanavatian, F. Zarein, F. Ataei, M. Nikkhah, P. Vandenabeele, Chem. Soc. Rev. **53**, 11557–11589 (2024). 10.1039/D3CS00743J 39417351 10.1039/d3cs00743j

[90] A. Pearce, T. Redfern-Nichols, E. Wills, M. Rosa, I. Manulak, C.Sisk, X.Huang, P. Atakpa-Adaji, D.L. Prole, G. Ladds, J. Cell Sci. **138**, JCS263434 (2025). 10.1242/jcs.263434 39810711 10.1242/jcs.263434 PMC11828474

[91] T. Azad, H.J. Janse van Rensburg, E.D. Lightbody, B. Neveu, A. Champagne, A. Ghaffari, V.R. Kay, Y. Hao, H. Shen, B. Yeung, B.A. Croy, K.L. Guan, F. Pouliot, J. Zhang, C.J.B. Nicol, X. Yang, Nat. Commun. **9**, 1061 (2018). 10.1038/s41467-018-03278-w 29535383 10.1038/s41467-018-03278-w PMC5849716

[92] Z. Claes, M. Bollen, Cell. Chem. Biol. **30**, 1666-1679.e6 (2023). 10.1016/j.chembiol.2023.07.018 10.1016/j.chembiol.2023.07.018 37625414

[93] Y. Gilad, R. Shiloh, Y. Ber, S. Bialik, A. Kimchi, Cell Rep. **8**, 909–921 (2014). 10.1016/j.celrep.2014.06.049 25066129 10.1016/j.celrep.2014.06.049

[94] H.-W. Yeh, O. Karmach, A. Ji, D. Carter, M.M. Martins-Green, H. Ai, Nat. Methods **14**, 971–974 (2017). 10.1038/nmeth.4400 28869756 10.1038/nmeth.4400 PMC5678970

[95] S. Iwano, M. Sugiyama, H. Hama, A. Watakabe, N. Hasegawa, T. Kuchimaru, K.Z. Tanaka, M. Takahashi, Y. Ishida, J. Hata, S. Shimozono, K. Namiki, T. Fukano, M. Kiyama, H. Okano, S. Kizaka-Kondoh, T.J. McHugh, T. Yamamori, H. Hioki, S. Maki, A. Miyawaki, Science **359**, 935–939 (2018). 10.1126/science.aaq1067 29472486 10.1126/science.aaq1067

[96] M.P. Hall, C.C. Woodroofe, M.G. Wood, I. Que, M. van’t Root, Y. Ridwan, C. Shi, T.A. Kirkland, L.P. Encell, K.V. Wood, C. Löwik, L. Mezzanotte, Nat. Commun. **9**, 132 (2018). 10.1038/s41467-017-02542-9 29317625 10.1038/s41467-017-02542-9 PMC5760652

[97] T. Kuchimaru, S. Iwano, M. Kiyama, S. Mitsumata, T. Kadonosono, H. Niwa, S. Maki, S. Kizaka-Kondoh, Nat. Commun. **7**, 11856 (2016). 10.1038/ncomms11856 27297211 10.1038/ncomms11856 PMC4911627

[98] Y. Su, J.R. Walker, Y. Park, T.P. Smith, L.X. Liu, M.P. Hall, L. Labanieh, R. Hurst, D.C. Wang, L.P. Encell, N. Kim, F. Zhang, M.A. Kay, K.M. Casey, R.G. Majzner, J.R. Cochran, C.L. Mackall, T.A. Kirkland, M.Z. Lin, Nat. Methods **17**, 852–860 (2020). 10.1038/s41592-020-0889-6 32661427 10.1038/s41592-020-0889-6 PMC10907227

[99] J. Chu, Y. Oh, A. Sens, N. Ataie, H. Dana, J.J. Macklin, T. Laviv, E.S. Welf, K.M. Dean, F. Zhang, B.B. Kim, C.T. Tang, M. Hu, M.A. Baird, M.W. Davidson, M.A. Kay, R. Fiolka, R. Yasuda, D.S. Kim, H.-L. Ng, M.Z. Lin, Nat. Biotechnol. **34**, 760–767 (2016). 10.1038/nbt.3550 27240196 10.1038/nbt.3550 PMC4942401

[100] K. Suzuki, T. Kimura, H. Shinoda, G. Bai, M.J. Daniels, Y. Arai, M. Nakano, T. Nagai, Nat. Commun. **7**, 13718 (2016). 10.1038/ncomms13718 27966527 10.1038/ncomms13718 PMC5171807

[101] M. Mirdita, K. Schütze, Y. Moriwaki, L. Heo, S. Ovchinnikov, M. Steinegger, Nat. Methods **19**, 679–682 (2022). 10.1038/s41592-022-01488-1 35637307 10.1038/s41592-022-01488-1 PMC9184281

[102] J.R. de Wet, K.V. Wood, D.R. Helinski, M. DeLuca, Proc. Natl. Acad. Sci. **82**, 7870–7873 (1985). 10.1073/pnas.82.23.7870 3906652 10.1073/pnas.82.23.7870 PMC390871

[103] Y. Nakajima, T. Yamazaki, S. Nishii, T. Noguchi, H. Hoshino, K. Niwa, V.R. Viviani, Y. Ohmiya, PLoS ONE **5**, e10011 (2010). 10.1371/journal.pone.0010011 20368807 10.1371/journal.pone.0010011 PMC2848861

[104] W.W. Lorenz, R.O. McCann, M. Longiaru, M.J. Cormier, Proc. Natl. Acad. Sci. **88**, 4438–4442 (1991). 10.1073/pnas.88.10.4438 1674607 10.1073/pnas.88.10.4438 PMC51675

[105] B.A. Tannous, D.-E. Kim, J.L. Fernandez, R. Weissleder, X.O. Breakefield, Mol. Ther. **11**, 435–443 (2005). 10.1016/j.ymthe.2004.10.016 15727940 10.1016/j.ymthe.2004.10.016

[106] S. Inouye, K. Watanabe, H. Nakamura, O. Shimomura, FEBS Lett. **481**, 19–25 (2000). 10.1016/S0014-5793(00)01963-3 10984608 10.1016/s0014-5793(00)01963-3

[107] S.V. Markova, S. Golz, L.A. Frank, B. Kalthof, E.S. Vysotski, J. Biol. Chem. **279**, 3212–3217 (2004). 10.1074/jbc.M309639200 14583604 10.1074/jbc.M309639200

